# The impact of bone marrow-derived mesenchymal stem cells on experimental testicular torsion in rats

**DOI:** 10.3906/sag-2105-168

**Published:** 2021-11-09

**Authors:** Ahmet ERTÜRK, Sabri DEMİR, Yasemin Dere GÜNAL, Mehmet ZENGİN, Miyase ÇINAR, Dinçer YILDIZ, Siyami KARAHAN, Emrah ŞENEL

**Affiliations:** 1 Department of Pediatric Surgery, Ankara City Hospital, Ankara, Turkey; 2 Department of Pediatric Surgery, Faculty of Medicine, Kırıkkale University, Kırıkkale, Turkey; 3 Department of Pathology, Faculty of Medicine, Kırıkkale University, Kırıkkale, Turkey; 4 Department of Biochemistry, Faculty of Veterinary, Kırıkkale University, Turkey; 5 Department of Anatomy, Faculty of Veterinary, Kırıkkale University, Kırıkkale, Turkey; 6 Department of Hystology, Faculty of Veterinary, Kırıkkale University, Kırıkkale, Turkey; 7 Department of Pediatric Surgery, Faculty of Medicine, Yıldırım Beyazıt University, Ankara, Turkey

**Keywords:** Testicular torsion, children, bone marrow-derived mesenchymal stem cells

## Abstract

**Background/aim:**

The aim of this study was to investigate the healing effects of bone marrow-derived mesenchymal stem cells (BM-MSCs) on experimental testicular torsion in rats.

**Materials and methods:**

Three groups consisting of 10 Wistar albino rats were created. In Group I, the left testicle was explored and relocated in the scrotum without any attempt to modify it. In Group II, the left testicle underwent torsion for three h and then was detorsed and relocated. In Group III, in addition to torsion and detorsion, BM-MSCs were administered intratesticularly. The rats were sacrificed on the seventh day, and the healing status of the testicles was investigated with histopathological and biochemical analyses. BM-MSC involvement was investigated by immunofluorescence microscopy. Statistical analysis was performed using SPSS 15.0. A p-value < 0.05 was considered statistically significant for all variables.

**Results:**

Immunofluorescence microscopy showed that BM-MSCs were located around the Leydig cells in Group III. Under light microscopy, the mean Johnsen Score of Group III was significantly higher than that of Group II (p = 0.035). The interleukin-10 (IL-10) level was significantly higher in Group III compared to Group II (p = 0.003). While the malondialdehyde (MDA) values in Group I (the control group) were lower than in the other groups (p = 0.037), the superoxide dismutase (SOD) values were similar (p = 0.158). Although there was no statistically significant difference between Group II and Group III in terms of MDA, it was lower in Group III. Although the tissue SOD levels were higher in Group III than in Group II, the difference was not statistically significant.

**Conclusion:**

This study has demonstrated that BM-MSCs significantly corrected the Johnsen Score and increased anti-inflammatory cytokine levels after testicular torsion. BM-MSCs can be used in testicular torsion as supportive therapy to minimize tissue damage.

## 1. Introduction

Testicular torsion can be simply defined as twisting of the spermatic cord. It has an estimated incidence of around 3.8 / 100,000 [1]. It is among the outstanding indications that require urgent surgical intervention in children. Although prevalent in all age groups, testicular torsion has a significant peak in adolescents and young men [2,3]. Both torsion and detorsion may create tissue damage by ischemia and reperfusion, respectively. It may concurrently cause structural and biochemical tissue alterations. Reperfusion damage is mostly related to increased free oxygen radicals and tissue infiltration by neutrophils. The resultant radicals cause peroxidation of the lipids in the cellular membrane, protein denaturation, and DNA damage [4]. Theoretically, reversal of ischemic damage, induction of spermatogenesis, and treatments that regulate immune reactions may potentially prevent the complications associated with testicular torsion [5].

Mesenchymal stem cells (MSCs) are multipotent stem cells that were initially isolated from bone marrow [6]. The main purposes of these cells are renewal and maintenance of the inhabited tissues. There is also evidence that they contribute to tissue and organ regeneration [7]. Thus, they have a critical role in wound healing [8].

MSCs can be isolated from bone marrow, fat tissue, skin, and umbilical cord blood [9]. When tissue is damaged, cytokines released from the damaged tissue and other factors induce MSCs in the bone marrow to proliferate, enter the circulation, and migrate to the target tissue [10]. They contribute to wound healing by residing in the damaged tissue. Allogenic use is possible as they are not immunogenic [11].

The suppressing effects of MSCs on inflammation and the immune response mediated by the release of high levels of interleukin-1 (IL-1) receptor antagonists has previously been established [12,13]. Additionally, these cells are notorious for being strong immune modulators, and potential therapeutic implications have been reported for acute ischemic disorders, such as acute myocardial infarction, stroke, traumatic brain damage, and acute liver failure [14–18].

The aim of this study was to investigate the impact of intratesticular application of bone marrow-derived mesenchymal cells (BM-MSCs) on the recovery process after detorsion.

## 2. Materials and methods

Thirty male Wistar albino rats were used in the study. Approval of the local ethical board for animal subjects was obtained. The rats were fed ad libidum.

### 2.1. Assignment of the subjects into groups

Three groups containing 10 rats each were created.

1. Group I (control group): The left scrotum of the rats was incised, and the left testis was dissected surgically. Then, the wound was repaired without any intervention.
2. Group II (torsion/detorsion [T/D] Group): The left testis was exposed surgically, and the testis was twisted 720°. After three h, the testis was untwisted, and the scrotum was repaired.
3. Group III (T/D + BM-MSC Group): Testicular torsion and detorsion were performed in the same way as in Group II. Immediately after detorsion, 5×10^4^ BM-MSCs stained with green fluorescent protein (GFP) were administered directly into the testis.

The rats were sacrificed, and blood and testicular tissue samples were obtained for comparison among the groups. Details of the groups are presented in Table 1.

### 2.2. Obtaining the BM-MSC isolates

The BM-MSC isolates were obtained from the Liv Hospital Regenerative Medicine and Stem Cell Production Center, İstanbul, Turkey. For MSC harvesting, the femur and tibial bones of the rats were opened, and the bone marrow was rinsed with Dulbecco’s Modified Eagle’s Medium (L-DMEM), which contains 10% fetal bovine serum (FBS) and 1% antibiotics (penicillin/streptomycin). The cells were filtrated with a 70 μm nylon filter, centrifuged for 10 min at 1800 rpm, diluted to 1/3 with phosphate-buffered saline (PBS), and spread over Histopaque-1077 (1.077 g/mL, Sigma–Aldrich, St. Louis, MO) for gradient centrifugation. Mononuclear cells were harvested after centrifugation and washed twice with PBS. The cells were incubated for three days in tissue culture plates with L-DMEM containing 1% antibiotics and 10% FBS in an environment of 37 °C and 5% CO_2_. The BM-MSCs could be isolated because of their ability to attach to the surface of the culture plates. Cells that could not attach were removed by changing the culture medium. After the cells reached a sufficient density, they were dissociated with 0.025% trypsin and passaged; they underwent characterization during the third passage.

### 2.3. Flow cytometric analysis

**Figure 1:**
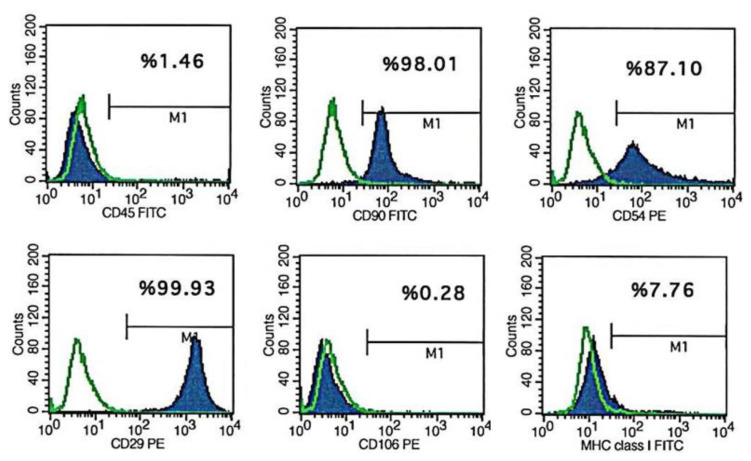
Histogram of flow cytometric analysis of BM-MSCs

The BM-MSCs were harvested on the third passage and underwent flow cytometric analysis with the FACS Calibur device (BD Biosciences, San Jose, CA). In this immunophenotyping, antibodies against CD29, CD45, CD54, CD90, CD106, and MHC Class I (BD Biosciences) were evaluated (Figure 1).

### 2.4. Labeling the cells with green fluorescent protein

In order to distinguish the intratesticularly administered BM-MSCs from the native MSCs of the rats, they were labeled with GFP. The cells were transfected with pGFP-N (Clontech, Palo Alto, CA) using the electroporation technique (Neon Transfection System, Invitrogen, Carlsbad, CA). After incubation for 48 h in L-DMEM with 10% FBS, the transfected cells were isolated by culturing with G418 (200 μg/mL). The BM-MSCs labeled with GFP were frozen to −80 °C in cryovials.

### 2.5. Testicular torsion/detorsion model and intratesticular administration of the labeled BM-MSCs

**Figure 2:**
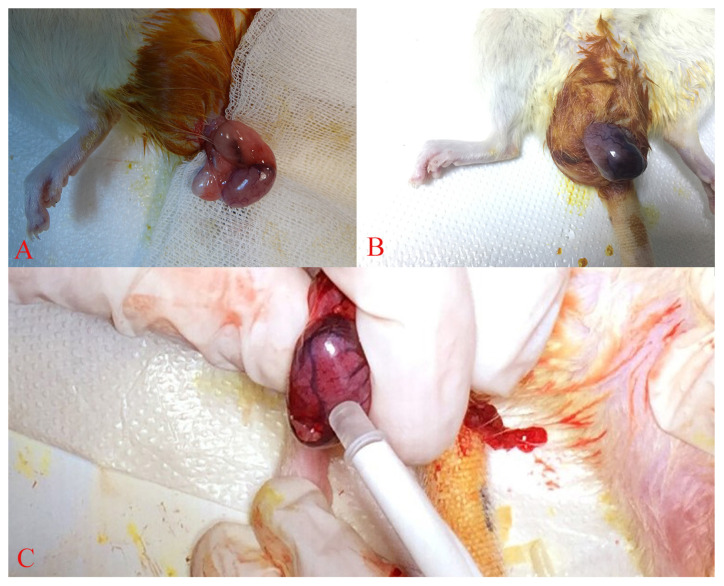
The stages of the study canbe seen in the rats. A) The left testes of the subjects in Groups II and III twisted 720° clockwise. B) Appearance of the testis 3 h after torsion. C) Intratesticular injection of 5×10^4^ BM-MSCs with micro insulin injector in Group III.

All procedures were performed under general anesthesia with intraperitoneal ketamine HCL (50 mg/kg; Ketalar 50 mg/mL, Pfizer) and xylazine HCl (5 mg/kg; Rompun 100 mg/mL, Bayer). Local surgical skin preparation was performed with 10% polyvinylpyrrolidone solution, and the left testis and spermatic cord were exposed with a scrotal incision in all subjects. In Group I, the testis was relocated to the scrotum, and the incision was closed without any further intervention. In Group II and Group III, the testis and spermatic cord were twisted 720° in a clockwise fashion (Figure 2A). To prevent unintended detorsion, the testis was fixed to the scrotum with silk sutures. The scrotal incision was repaired, and the rats were kept in their cages for three hours.

After three hours, general anesthesia was induced again, and the twisted testis was untwisted through the same incision (Figure 2B). In Group II, the untwisted testis was relocated in the scrotum, and the scrotal incision was repaired with silk sutures. In Group III, 5×10^4^ BM-MSCs were administered intratesticularly under sterile conditions after untwisting of the testis (Figure 2C). To protect the GFP-labeled cells from light, microinsulin injectors were draped with lightproof foil. After completion of the injection, the testis was relocated in the scrotum, and the scrotum was repaired with silk sutures. During the procedure, all rats received intraperitoneal Ringer’s Lactate solution (5 mL/kg/h) for fluid maintenance. All rats also received 100% O_2_ until they recovered from anesthesia. All rats were sacrificed on the seventh day after blood samples were drawn for biochemical analyses, and the left testes were excised for histopathological and biochemical evaluation.

### 2.6. Histopathological evaluation of the testes

Testicular tissue samples were initially stained with hematoxylin and eosin (H&E). Spermatogenic functions were evaluated with a light microscope using the Johnsen score [19]. In Group III, tissue sections were stained with GFP antibody (fluorescein isothiocyanate [FITC]; catalog code: ab6662, Santa Cruz Biotechnology Inc., Dallas, TX) for evaluation of the labeled BM-MSCs in the testicular tissue with an immunofluorescent microscope.

### 2.7. Biochemical analysis

The blood samples were centrifuged for 10 min at 4°C with 3000 rpm for serum acquirement and stored at −80°C until analyses. Serum testosterone levels were measured with a commercially available kit (Cayman, CA. No:582701, USA) using a microplate reader (Thermo Scientific Multiskan).

The blood of the tissue samples was removed by immediately rinsing with distilled water and 0.9% saline solution, and the samples were stored in Eppendorf tubes wrapped with aluminum foil at −80°C until the tests were performed. Testicular tissue samples (0.15–1 g) were stored in test tubes containing a buffer solution with a pH value of 7.4 and homogenized in ice for one minute with an ultrasonic homogenizer (Bandelin, Germany). The homogenized products were centrifuged for 20 min at 4 °C with 1600 rpm speed, and the supernatants that were produced were stored at −20 °C until the analyses were performed. Finally, the supernatants were centrifuged for 10 min at 3000 rpm before the analyses were performed. The supernatants were analyzed for malondialdehyde (MDA), tumor necrosis factor alpha (TNF-α) (Shanghai Sunred Biological Technology Co., Ltd, Shanghai, China), interleukin-6 (IL-6) (SunRed), and interleukin-10 (IL-10) (SunRed) levels in addition to superoxide dismutase (SOD) (Cayman, USA) activity with commercial test devices (SunRed) using a microplate reader (Thermo Scientific Multiskan). MDA levels were measured with the method described by Buege and Aust in 1978 [20].

### 2.8. Statistical analysis

Statistical analyses were performed using the Statistical Package for the Social Sciences (SPSS) version 15.0 software. The normality of the Johnsen scores of the histopathological results was evaluated with the Shapiro–Wilk test. Descriptive statistics were expressed with mean ± standard deviation. The Kruskal–Wallis test was used for comparison of the groups. A p-value < 0.05 was considered statistically significant.

For statistical analysis of the biochemical results, a preliminary evaluation was performed to determine whether the parametric test hypothesis (distribution of normality and homogeneity of variance) was met. The normality of the data was evaluated with the Shapiro–Wilk test and found to be distributed normally. One-way analysis of variance was used to identify the significance of the difference of mean values and for statistical analysis of the groups. The Duncan test was used to identify the significance of differences among the groups. Data were expressed as mean values and standard error of the mean values (X ± SE).

## 3. Results

### 3.1. Characterization of the BM-MSCs

After the third passage of the BM-MSCs, flow cytometric analysis performed with Cell Quest (BD Biosciences) software revealed positive results for CD29, CD54, CD90, and MHC Class I antibodies and negative results for CD45 and CD106 antibodies. Characterization of the BM-MSCs was performed depending on these immunophenotyping studies.

### 3.2. Evaluation of the tissue specimens under light microscopy

**Figure 3:**
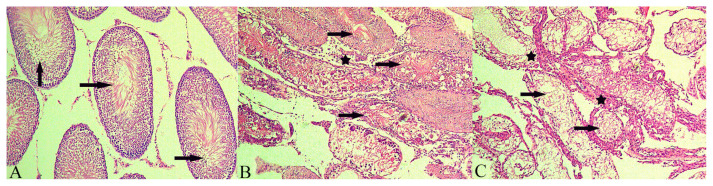
Images of the testes under light microscope (H&E, X200 magnification). a) Histological sections of testes in Group I. Arrow indicates normal spermatogenesis in the seminiferous tubules. b) Histological sections of testes in Group II. Arrow indicates spermatogonium, star sign indicates Leydig cell hyperplasia. c) Histological sections of testes in Group III. Arrow indicates spermatocytes, star sign indicates Leydig cell hyperplasia.

Light microscope evaluation of the specimens after H&E staining demonstrated histological findings of normal testicular tissue in Group I (Figure 3A). In group II, germ cell maturation was arrested in the spermatogonia phase, and scattered areas of Leydig cell hyperplasia were observed (Figure 3B). In Group III, Leydig cell hyperplasia was more common than in Group II, but germ cells were observed to reach the spermatocide phase (Figure 3C).

### 3.3. Comparison of the Johnsen scores

The Johnsen scores were calculated depending on the histopathological appearance of the testicular tissues, and mean values were obtained in each group (Table 2). One rat in Group II had a respiratory problem during recovery from the anesthesia and was found dead in its cage one day later. Accordingly, the mean values were calculated for the remaining nine rats in Group II. The mean Johnsen score of Group III was significantly higher than that of Group II (p < 0.035), while the mean Johnsen score of Group I was significantly higher than Groups II and III (p < 0.001) (Table 3).

### 3.4. Examination of tissue specimens that received BM-MSCs with immunofluorescent microscopy

**Figure 4:**
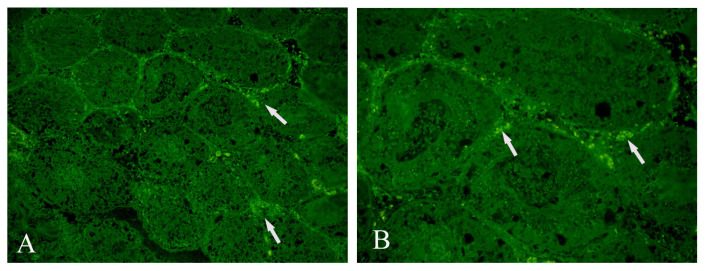
Image of stem cells (indicated by arrow) labeled with green fluorescent protein (GFP) condensed around the tubules under the IF microscope (A: X200, B: X400).

Immunofluorescent examination of the specimens in Group III revealed dense accumulation of GFP-stained BM-MSCs around the seminiferous tubules (Figures 4A and 4B).

### 3.5. Biochemical results

Although the IL-10 levels in Group III were not significantly different from Group I, they were significantly higher than in Group II (p = 0.003). Although none of the groups demonstrated a significant difference in TNF-α levels, they were slightly lower in Group III than in Group II (1148.91 ± 150.17 versus 1287.34 ± 129.14, respectively; p = 0.207). No significant difference was observed among the groups for tissue IL-6 levels (p = 0.472).

MDA levels were not significantly different between Groups I and III but were significantly higher in Group II than in Group I (p = 0.037). Although not statistically significant, MDA levels were found to be lower in Group III than in Group II (7.72 ± 1.06 and 8.80 ± 0.86, respectively). Although no statistically significant difference was found, tissue SOD levels were higher in Groups I and III compared to Group II (88.49 ± 16.18, 87.56 ± 8.87, and 60.59 ± 9.56, respectively; p = 0.158). Testosterone levels were not significantly different among the groups (p = 0.418).

Statistical analyses of the biochemical test results are shown in detail in Table 4.

## 4. Discussion

The results of this study demonstrated that the Johnsen score was higher in rats that received intratesticular BM-MSCs than in those that did not. Additionally, IL-10, which is a well-known anti-inflammatory cytokine, levels were found to be higher in the rats that received BM-MSCs.

Testicular torsion is a serious condition that may cause testicular damage and even result in loss of the testis if not recognized and treated promptly. The resultant testicular damage depends on the degree of torsion and the time between the onset of symptoms and surgical intervention. Unfortunately, early recognition and surgical intervention is the only parameter that surgeons have an impact on. Accordingly, early surgical intervention is essential, and in the case of strong clinical suspicion, immediate surgery is recommended to avoid losing precious time with imaging studies [21]. In previous experimental studies focusing on reducing or preventing ischemia / reperfusion damage, the intervention was generally performed before detorsion [5,21–23]. By doing so, they intended to prevent the reperfusion damage that occurs after detorsion. The definitive diagnosis of many conditions that may be confused with testicular torsion (epididymitis, epididymo-orchitis, and torsion of the testicular appendages) can be achieved after surgical exploration in children. In order to simulate real-life clinical conditions, we administered BM-MSCs after surgical correction of the torsion and exclusion of other conditions. The main purposes of this approach were to prevent unnecessary utilization of BM-MSCs and invasive interventions. Although many studies have focused on testicular torsion, not many have evaluated the impact of BM-MSC treatment.

The Johnsen score, which was described by Svend G. Johnsen in 1970, is a common and well-known histological index that investigates spermatogenesis. This index mainly evaluates the absence of the most mature cell type of spermatogenesis, which indicates testicular damage and progressive degeneration of germinal epithelium. In a torsion model similar to our study, Hsiao et al. found higher Johnsen scores in rats that received orbital fat tissue-derived MSCs administered before testicular torsion [5]. In our study, the BM-MSCs administered after detorsion in Group III were found to accumulate in the seminiferous tubules and around Leydig cells. As the Johnsen score in this group was found to be higher and there was formation of dense cellular aggregates, we propose that BM-MSC application improves spermatogenesis in testicular torsion.

Functional Leydig and Sertoli cells are necessary for undisturbed spermatogenesis. Leydig cells reside in the interstitial spaces adjacent to the seminiferous tubules. In the presence of luteinizing hormone, they produce testosterone. Testosterone and follicle-stimulating hormone induce differentiation of spermatogonia by inducing Sertoli cells, which is essential in spermatogenesis [24]. Reduction in blood testosterone levels after testicular torsion has previously been observed by other researchers. Apoptosis in germ cells and Leydig cell dysfunction were held responsible for this observation [25]. We also observed that testosterone levels were reduced after torsion, and even BM-MSC application did not prevent this reduction.

Ischemia secondary to torsion has a negative impact on steroidogenesis by Leydig cells and spermatogenesis. After ischemia, plasma levels of proinflammatory cytokines, such as TNF-α, IL-1, and IL-6, increase significantly and cause exacerbation of tissue damage [26]. Additionally, MDA levels increase after ischemia, which is the end product of lipid peroxidation and a common marker of oxidative stress. Increased MDA levels indicate increased free oxygen radicals [27,28]. It is a common judgement that free oxygen radicals are responsible for the tissue damage that occurs secondary to the ischemia-reperfusion process. There are many antioxidant defense mechanisms to clear free oxygen radicals. SOD, which is present in most cell types, increases the elimination of free oxygen radicals by catalytic processes [29]. On the other hand, anti-inflammatory cytokines protect damaged tissues by suppressing proinflammatory cytokine production after ischemia. One of these anti-inflammatory cytokines is IL-10, a well-known modulator of inflammatory reactions that acts by inhibiting proinflammatory cytokines like TNF-α, IL-1, IL-6, and IL-8. Furthermore, IL-10 has been reported to protect endothelial function after an inflammatory stimulus [30,31]. Some authors have reported that BM-MSCs migrate to damaged tissue and inhibit the immune and inflammatory response by their anti-inflammatory and immunomodulatory properties, which facilitate repair of the damaged tissue. In addition, the antioxidant properties of BM-MSCs have also been reported [32,33]. In our study, although it did not reach statistical significance, reduced MDA and TNF-α levels and increased SOD levels were found in the rats that received BM-MSCs. In accordance with the literature, BM-MSCs were found to increase IL-10 levels [24,34,35]. The results of our study confirm the anti-inflammatory and antioxidant properties of BM-MSCs, which is consistent with the literature [17,34,36,37].

The major limitation of this study is that it was an experimental animal study. Since the physiological and anatomical characteristics of any animal do not match humans, human studies are needed to confirm or refute our findings. The second limitation is the lack of long-term results. Long-term follow-up and results are necessary in order to evaluate the impact of BM-MSCs on the long-term complications of torsion (including infertility and azoospermia). The final limitation of this study is that the amount of BM-MSCs needed to be administered per tissue weight is unclear. We used similar amounts to those used in previous studies.

In conclusion, testicular torsion is a serious condition in pediatric surgery and urology practice because of its significant sequelae and complications. It may cause testicular loss and infertility despite early surgical intervention. Our results suggest that BM-MSC treatment improves the recovery of spermatogenesis and reduces the negative impact of the oxidative stress process when applied after detorsion in testicular torsion confirmed by surgical exploration. Thus, BM-MSC treatment should be considered as an adjunctive measure in patients with surgically confirmed testicular torsion. As allogenic utilization is possible, BM-MSCs harvested from healthy volunteers can be used as an adjunctive treatment in testicular torsion, providing appropriate replication and storage. Human clinical studies are necessary to confirm these findings.

## Figures and Tables

**Table 1:** Formation of experimental groups.

Groups	Detail of the groups	Number of rats
**Group I**	Control group	10
**Group II**	Group of torsion/detorsion in the left testis (T/D)	10
**Group III**	Group that BMMSCs were given immediately after torsion/detorsion in the left testicle	10
	**Total**	**30**

**Table 2:** Johnsen Scores of the rats.

	Group I		Group II		Group III	
Rat number	Johnsen Score	Mean; Std.dev.	Johnsen Score	Mean; Std.dev.	Johnsen Score	Mean; Std.dev.
1	10	9.7 ± 0.48	2	3.33 ± 1.11	7	5.3 ± 1.82
2	10		2		8	
3	9		3		3	
4	9		4		4	
5	10		2		3	
6	10		4		5	
7	10		4		4	
8	9		4		5	
9	10		5		7	
10	10		Deceased		7	

**Table 3:** Statistical comparison of Johnsen scores of subjects.

Variable	Group I	Group II	Group III	p*	p**
**Johnsen scores (Mean; Std.dev.)**	9.7 ± 0.48	3.33 ± 1.11	5.3 ± 1.82	0.035	<0.001

p* Johnsen scores of rats in Group II and Group III were compared. Mann–Whitney U test used.

p** Johnsen scores of rats in Group I, Group II, and Group III were compared. Kruskal–Wallis test used.

**Table 4:** Statistical comparison of left testicular tissue cytokines (TNF-α, IL-10, IL-6), malondialdehyde (MDA) and superoxide dismutase (SOD), and blood testosterone levels of the groups.

Variables	Group I (Mean;Std.Dev)	Group II (Mean;Std.Dev)	Group III (Mean;Std.Dev)	*p* *
TNF-α (pg/GR-tissue)	897.64 ± 166.49	1287.34 ± 129.14	1148.91 ± 150.17	0.207
IL-10 (pg/GR-tissue)	631.23 ± 127.61^a^	283.36 ± 12.40^b^	635.81 ± 49.15^a^	0,003**
IL-6 (pg/GR-tissue)	467.80 ± 88.47	571.35 ± 82.91	621.16 ± 90.05	0,472
MDA (μmol/GR-tissue)	5.25 ± 0.71^b^	8.80 ± 0.86 ^a^	7.72 ± 1.06 ^ab^	0.037**
SOD (U/GR-tissue)	88.49 ± 16.18	60.59 ± 9.56	87.56 ± 8.87	0.158
Testosterone (pg/mL)	535.54 ± 71.64	430.73 ± 72.8	408.18 ± 36.8	0.418

* ANOVA test used.

** Duncan test used as post-hoc test. Groups carrying different letters in the same line are statistically different from each other.
